# Treatment of splenic flexure colon cancer: a comparison of three different surgical procedures: Experience of a high volume cancer center

**DOI:** 10.1038/s41598-019-47548-z

**Published:** 2019-07-29

**Authors:** Daniela Rega, Ugo Pace, Dario Scala, Paolo Chiodini, Vincenza Granata, Andrea Fares Bucci, Biagio Pecori, Paolo Delrio

**Affiliations:** 1 Colorectal Surgical Oncology, Abdominal Oncology Department, Istituto Nazionale per lo Studio e la Cura dei Tumori, “Fondazione G. Pascale” IRCSS, Naples, 80131 Italy; 2 Medical Statistics Unit, University of Campania “Luigi Vanvitelli”, Naples, 80138 Italy; 3 Radiology Unit, Dipartimento di Supporto ai Percorsi Oncologici Area Diagnostica, Istituto Nazionale per lo Studio e la Cura dei Tumori, “Fondazione G. Pascale” IRCSS, Naples, 80131 Italy; 4 Radiotherapy Unit, Istituto Nazionale per lo Studio e la Cura dei Tumori, “Fondazione G. Pascale” IRCSS, Naples, 80131 Italy

**Keywords:** Colon cancer, Colon cancer, Surgical oncology, Surgical oncology

## Abstract

Extended right or left hemicolectomy are the most common surgical treatments for splenic flexure colon cancer. Extended resection (including distal pancreasectomy and/or splenectomy), has been often indicated for the treatment for the splenic flexure cancer, because the lymphatic drainage at this site is poorly defined and assumed as heterogeneous. Between January 2006 and May 2016, 103 patients with splenic flexure colon cancer were enrolled in the study. We evaluated the clinicopathological findings and outcomes of all patients and associated them to the different surgical treatment. Out of 103 selected cases an extended right hemicolectomy was performed in 22 (21.4%) patients, an extended left hemicolectomy in 24 (23.3%) patients, a segmental resection of the splenic flexure in 57 (55.3%) patients; the combined resection of adjacent organs showing tumor adherence was carried out in 11 (10.7%) patients. The tumor infiltrated near organs (T4) in 5 patients. No significant differences in complications were found among the three groups. In all groups no differences were found in the total number of harvested lymphnodes. After a median follow-up of 42 months, 30 recurrences and 19 deaths occurred (12 for tumor progression). There was no difference in overall and progression free survival among the three different surgical treatments. According to our results, the partial resection of splenic flexure was not associated with a worse prognosis and it was leading for a satisfactory oncological outcome. It is our opinion that the extended surgery is seldomly indicated to cure splenic flexure cancer.

## Introduction

Splenic flexure cancer (SFC) is defined as a colon cancer situated in the distal third of the transverse colon, or in the left colonic corner, or in the proximal descending colon within 10 cm from the flexure^1^. It is relatively rare and represent only 1–8% of all colon cancers^2,3^.

The diagnosis of this cancer is often late, in an advanced stage of illness, mainly with an obstructive clinical presentation^4^.

Pathologic stage currently remains the main prognostic factor, although it cannot fully predict the clinical outcome by itself. Other factors contribute to the prognosis namely histological grade of differentiation, presence of lymphovascular and perineural invasion, the quality of the specimen with complete mesocolic excision (CME), the presence of perforation or intestinal obstruction at the time of diagnosis.

A standard surgical approach to splenic flexure cancer has not been described and various extent of resections have been advocated, going from extended colectomy to segmental resection, with or without adjacent organ resections (i.e. spleen or distal pancreas). As matter of fact, the challenge is related to the peculiar dual lymphatic drainage of the superior and inferior mesenteric vessels, which lie between the right and left territories^3,5,6^.

To date, several authors have evaluated the differences between laparoscopic and open approach, or have compared intracorporeal versus extracorporeal anastomosis^7,8^. At the best of our knowledge, only few studies investigated a segmental splenic flexure resection compared to extended colectomy, with the goal of proving the oncological equivalence between these two approaches. Moreover only the 14% of surgeons seem to perform segmental resection for splenic flexure cancer^6,9–11^.

In order to improve the knowledge about the outcome of different procedures, we have compared the results of three different approaches (extended right hemicolectomy, extended left hemicolectomy and segmental resection) to splenic flexure cancer, examining the pathologic and oncologic outcomes in patients operated with curative intent in our Institution and prospectively included into a dedicated colorectal cancer database.

## Materials and Methods

In order to compare the three different surgical techniques, we assessed a previous study^12^, and searched the surgical database of the Istituto Nazionale del Tumori di Napoli (IRCCS Fondazione G. Pascale) from January 2006 to May 2016.

During this period, 1408 surgical resections for colorectal cancer were performed. Of these, 120 (8.5%) patients who underwent resection of splenic flexure cancer were selected.

**Figure 1:**
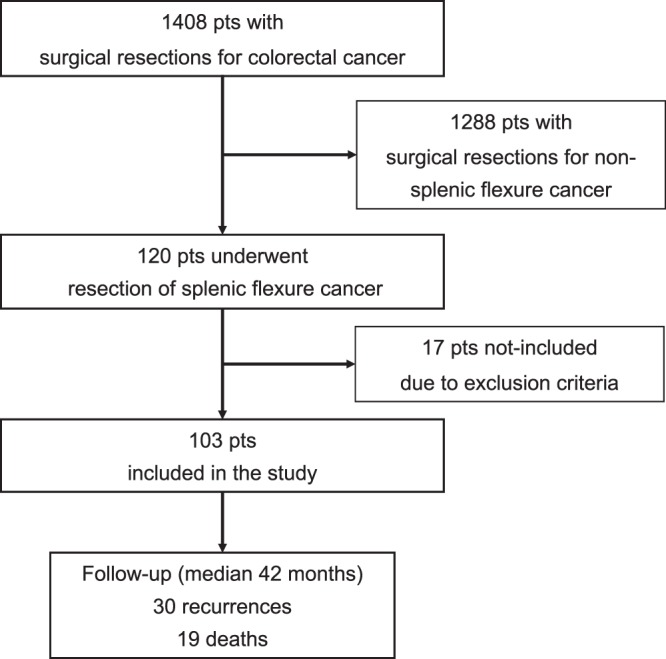
Flowchart of the study.

We excluded patients with metastasis, metachronous or synchronous colorectal cancers, R1 resection on final pathological report, colic polyposis and patients operated on for benign lesions. Moreover, we excluded patients undergoing palliative resection and/or emergency surgery because of perforation or acute obstruction. Finally 103 patients who underwent curative resection of a splenic flexure cancer were included. A flowchart of the study was reported in Fig. 1.

The routine preoperative evaluations included physical examination, serum carcinoembryonic antigen (CEA), colonoscopy with biopsy, whole body computed tomography (CT). The CT scan was integrated to a virtual colonoscopy in cases of incomplete endoscopic exam. Preoperative endoscopic tattooing was performed if cases of unclear cancer location and in patients with planned laparoscopic approach.

Patient characteristics were registered as follows: age, sex, American Society of Anesthesiologists (ASA) score, year of surgery, surgical approach, kind of resection, operative details, histological results, postoperative outcomes and oncological follow-up results. Postoperative surgical outcomes included complication and mortality rate within 30 days from the surgery. Postoperative complications were graded according to the Clavien classification^13,14^, considering class I and II as less relevant, and class III, IV and V as truly significant.

As for the pathologic report, the number of harvested lymphnodes and the presence among them of positive ones were considered. The disease stage was evaluated according to the American Joint Commitee on Cancer Staging, 7th Edition^15^.

Patients were then divided into three groups according to the type of resection: extended right hemicolectomy (ERH), extended left hemicolectomy (ELH) and segmental splenic flexure resection (SSFR).

All patients with stage III tumours or with unfavorable histopathological characteristics underwent adjuvant chemotherapy, unless contraindications. Chemotherapy regimes were based on the guidelines of the Italian Medical Oncology Association – Associazione Italiana Oncologia Medica - AIOM^16^.

The patients were followed-up postoperatively every 4 months for the first 2 years, every 6 months for the next 3 years, then annually. The evaluations included physical examination, serum carcinoembryonic antigen (CEA); whole body CT scan and colonoscopy were performed annually. Patients with suspected recurrence or metastases underwent specific examinations (e.g. PET-CT, MRI, bone scans, biopsy). Locoregional recurrence was defined as biopsy proven carcinoma of the bowel wall or within the lymphatic drainage area in the region of primary tumor. Distant recurrence was defined as recurrent tumors in the peritoneum, liver, non-regional lymph nodes or locations outside the abdominal cavity, such as lung and bones, confirmed by imaging and/or pathological examination. As for chemotherapy, adopted follow up protocols were based on the guidelines of the Italian Medical Oncology Association – Associazione Italiana Oncologia Medica - AIOM^16^.

Progression free survival has been defined as the time from surgery to disease recurrence. Overall survival was defined as the time from surgery to death.

### Surgical technique

No patient received mechanical oral bowel preparation. All patients received perioperative antibiotic and antithrombotic prophylaxis.

Extended right hemicolectomy is defined as the resection of the right and transverse colon and a part of descending colon along with together regional lymph nodes. The ileocolic, the right colic (if present), the middle colic and the left colic vessels were ligated at their origins. Usually an ileocolic mechanical end-to-side anastomosis was performed.

Extended left hemicolectomy was defined as the resection of the colonic segment between the left third of the transverse colon and the colorectal junction. The inferior mesenteric vessels and the left branch of the middle colic vessels were ligated at their origins, and a regional lymphadenectomy was performed. Intestinal continuity was restored by a side-to-end mechanical colorectal anastomosis^9,17,18^.

Segmental splenic flexure resection was defined as resection of the colonic segment located between the distal transverse colon and the first descending segment of the colon. In this case, the left branch of middle colic and the left colic vessels were ligated at their origin together with the inferior mesenteric vein^19^. Usually a mechanical side-to-end colo-colic anastomosis was performed. When technically feasible the resection of the colon was performed with the entire regional mesocolon in an intact peritoneal package^20^.

The extended (right or left) resection was usually indicated with one or more of following conditions identified with the support of the radiologist at the preoperative evaluation: T4 stage, metastatics regional lymphnodes, clear vascular involvement, large tumour size. The preoperative plan was then confirmed intraoperatively and usually related to the actual site of the tumour (distal third of the transverse colon - ERH versus proximal descending colon - ELH). A ERH was also indicated in cases of proximal dilated colon. According to renown guidelines in case of adjacent organ involvement for malignant or inflammatory adhesions, an en-bloc resection was performed^16,21^.

The laparoscopic approach was usually performed by surgeons with larger experience in minimally invasive surgery whether an open approach was usually dedicated to patients with more extended disease, a suspected invasion of other organs, this was usually defined during the multidisciplinary meeting with the support of the radiologist in charge. A suspected infiltration of the pre-renal fat or of the pancreatic tail were an usual indication to an open operation.

Our institutional ethics committee approved this retrospective study.

### Statistical analysis

Continuous variables were reported as either mean and standard deviation (SD) or median and range on the basis of their distribution. Comparisons of variables among groups were performed by the one-way ANOVA or Kruskal–Wallis test. Categorical variables were expressed as the absolute number and percentage and analyzed by the Chi-square test. When appropriate exact test was calculated. Median follow-up was estimated by inverse Kaplan-Meier approach. Survival curves were estimated by the product-limit method of Kaplan-Meier and compared by the log-rank statistic. Cox regression model was used to estimate adjusted association between surgical treatments and survival endpoints. Covariates were included *a priori* in the Cox regression models. A two-tailed p-value < 0.05 was considered significant. Data were analyzed using SAS 9.2 (SAS Institute Inc., Cary, NC, USA) and R software version 3.3.3 (R Foundation for Statistical Computing, Vienna, Austria).

### Ethical approval

All procedures performed in studies involving human participants were in accordance with the ethical standards of the institutional and/or national research committee and with the 1964 Helsinki declaration and its later amendments or comparable ethical standards.

### This is a retrospective study

For this type of study formal consent is not required.

## Results

A total of 103 patients treated with surgery for splenic flexure cancer were included in the study: 22 (21.4%) underwent extended right hemicolectomy, 24 (23.3%) underwent extended left hemicolectomy, and 57 (55.3%) underwent segmental splenic flexure resection. Baseline characteristics of patients were reported in Table 1. 58.3% of the patients were male, with a mean age of 65.8 years (SD 10.0 years). ASA score 2 and 3 were predominant (37.9% and 59.2% respectively). The tumor lesion was substenotic in 31.1% of cases. No differences were found among three groups in the baseline characteristics, except for a significant difference in the substenotic lesion, which appeared with higher prevalence in the SSFR group (p = 0.043).

**Table 1:** Characteristics at baseline.

	ERH	ELH	SSFR	p-value
	(n = 22)	(n = 24)	(n = 57)	
Age, year, mean (SD)	65 (7.5)	63.8 (10.9)	67 (10.5)	0.380
Male gender, n (%)	13 (59.1)	13 (54.2)	34 (59.7)	0.897
ASA Score, n (%)				0.462
1	1 (4.6)	0 (0.0)	0 (0.0)	
2	7 (31.8)	9 (37.5)	23 (40.4)	
3	14 (63.6)	15 (62.5)	32 (56.1)	
4	0 (0.0)	0 (0.0)	2 (3.5)	
5	0 (0.0)	0 (0.0)	0 (0.0)	
Substenotic lesion, n (%)	6 (27.3)	3 (12.5)	23 (40.4)	0.043

Mean operative time was 121′ in the ERH group, 109′ in the ELH group and 105′ in the SSFR group. The laparoscopic approach was used in 17.5% of patients (13.6% in the ERH group, 20.8% in the ELH group, 17.5% in the SSFR group). Multiorgan resection was performed in 10.7% of patients (4.6% in the ERH group, 8.3% in the ELH group, 14.0% in the SSFR group). There were no perforations of the cancer or violation of the tumor during the procedures. No differences were found among three groups in the surgery characteristics. Characteristics of surgery were reported in Table 2.

**Table 2:** Surgery and post-surgery characteristics.

	ERH	ELH	SSFR	p-value
	(n = 22)	(n = 24)	(n = 57)	
Operative time, mean (SD)	121′ (58.6′)	109′ (50.8′)	105.3′ (49.6′)	0.484
Laparoscopic approach, n (%)	3 (13.6)	5 (20.8)	10 (17.5)	0.833
Multiorgan Resection, n (%)	1 (4.6)	2 (8.3)	8 (14.0)	0.486
Hospitalization (day), mean (SD)	7.9 (3.7)	8 (3.2)	6.9 (3.1)	0.249
Clavien Score, n (%)				0.851
1–2	20 (90.9)	23 (95.8)	53 (93.0)	
3	2 (9.1)	1 (4.2)	3 (5.3)	
4	0 (0.0)	0 (0.0)	0 (0.0)	
5	0 (0.0)	0 (0.0)	1 (1.8)	

### Postoperative outcomes

Mean length of hospital stay was 7.4 days (SD 3.3 days), with 7.9, 8 and 6.9 days for the ERH, ELH and the SSFR group, respectively.

The postoperative outcomes were uneventful or with less severe complications (Clavien I–II) in 96 patients (93.2%).

Sever postoperative complications (Clavien III–V) occurred in 7 patients (6.8%). Three patients developed a significant anastomotic leak requiring a reoperation; in one patient a splenectomy was necessary owing to a splenic postoperative bleeding; one patient was treated with Vacuum Assisted Closure (VAC) therapy for a severe wound complication; one patient developed a large intraabdominal collection that was drained under radiological assistance; one patient died of intestinal ischemia with a 30-days mortality rate 1.0%.

No significant differences in complications according to severity, reoperation rate, hospital stay, 30-day mortality, were observed in the three groups.

Postoperative characteristics were reported in Table 2.

### Histopathological staging

Tumor-free resection margin was reported in all specimens and tumor distance from proximal and distal margins was always adequate. A pT4 tumor were found in only 4.8% No differences were found among three groups in the pTNM and stage values. Carcinoma of moderate differentiation (G2) was present in 84 patients of patients. The mean number of harvested lymphnodes was 23.5 and a significant difference in total number of harvested lymphnodes was found among groups, whit a higher mean in the ERH group (p = 0.040). No differences were found among three groups regarding the correct lymphoadenectomy, being the total number of harvested lymphnodes larger than 12 in all group (p = 0.463). Histopathologic characteristics were reported in Table 3.

**Table 3:** Histophatologic Characteristics.

	ERH	ELH	SSFR	p-value
	(n = 22)	(n = 24)	(n = 57)	
Lymphnodes harvested, mean (SD)	28.9 (13)	23.3 (13.9)	21.5 (9.6)	0.040
number ≥12, n (%)	21 (95.5)	20 (83.3)	50 (87.7)	0.463
T, n (%)				0.790
1	4 (18.2)	7 (29.2)	15 (26.3)	
2	7 (31.8)	3 (12.5)	10 (17.5)	
3	10 (45.5)	13 (54.2)	29 (50.9)	
4	1 (4.6)	1 (4.2)	3 (5.3)	
N, n (%)				0.754
0	16 (72.7)	15 (62.5)	38 (66.7)	
1	2 (9.1)	5 (20.8)	12 (21.1)	
2	4 (18.2)	4 (16.7)	7 (12.3)	
Stage, n (%)				0.891
I	9 (40.9)	10 (41.7)	23 (40.4)	
IIA	6 (27.3)	3 (12.5)	14 (24.6)	
IIC	1 (4.6)	1 (4.2)	1 (1.8)	
IIIA	1 (4.6)	0 (0.0)	1 (1.8)	
IIIB	3 (13.6)	8 (33.3)	12 (21.1)	
IIIC	2 (9.1)	2 (8.3)	6 (10.5)	
G3-V1-mucinosus type, n (%)	3 (13.6)	2 (8.3)	17 (29.8)	0.060

### Oncologic outcomes

**Figure 2:**
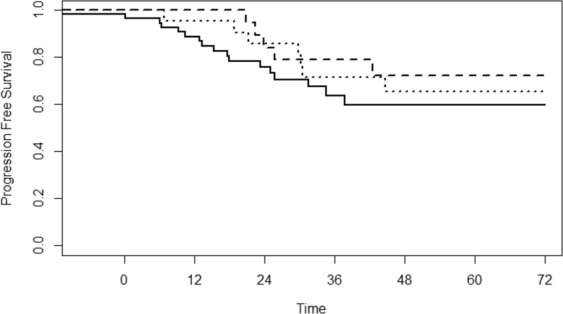
Progression free survival curves by surgical treatment groups. Solid line: segmental splenic flexure resection (SSFR), dashed line: extended left hemicolectomy (ELH), dotted line: extended right hemicolectomy (ERH).

**Figure 3:**
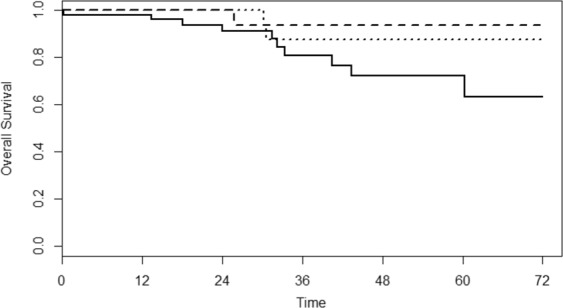
Overall survival curves by surgical treatment groups. Solid line: segmental splenic flexure resection (SSFR), dashed line: extended left hemicolectomy (ELH), dotted line: extended right hemicolectomy (ERH).

The median follow up was 42 months (IQR 24–70 months). Local recurrence was found in 2 patients (both in the SSFR group), distant recurrence in 21 patients (5 in the ELH group, 4 in the ERH group, 12 in the SSFR group). During follow-up, 30 recurrences and 19 deaths occurred (12 for tumor progression). No statistically significant differences were found for progression free survival (Fig. 2, p = 0.621) and overall survival (Fig. 3, p = 0.211) among the three groups. Similar results were obtained adjusting for stage and for undifferentiated grading (G3) or vascular infiltration (V1) or mucinosus type, using Cox regression model with no significant difference among the three groups for progression free survival (p = 0.879) and overall survival (p = 0.328).

## Discussion

The optimal surgical approach for splenic flexure cancer has not been clearly established and it is debated, mainly for the incomplete understanding of the peculiar dual lymphatic drainage of this region, related to the superior and inferior mesenteric vessels^3,5,6^.

A pioneering study from Griffith described that the splenic flexure is supplied by terminal branches of the left colic artery in 89% of cases and by the superior mesenteric artery through the middle colic in 11%. The middle colic artery was observed to be missing in the 22% of cases^22^.

By histological examination Nakagoe and coworkers demonstrated that the majority of metastatic lymphnodes are located along the paracolic arcade and the left colic artery^23^.

Vasey *et al*. studied lymphatic drainage of splenic flexure by intraoperative scintigraphic mapping and they concluded that this is preferentially directed toward the left colic in the 96% of patients^24^.

Some authors believe that an extended (right or left) hemicolectomy is better indicated to guarantee the removal of all potentially involved lymphnodes along the superior mesenteric vessels^6,8,25^. They consider the segmental resection less radical than extended hemicolectomy and usually perform the former in older patients or for palliation in cases of extensive disease^26^.

In contrast, other Authors^2,3,10^ reported that the dual lymphatic drainage did not confer a survival disadvantage and extended resection was unnecessary.

A correct oncological lymphoadenectomy in colorectal cancer should involve the removal of at least 12 lymphnodes in the surgical specimen^27,28^. This, associated to the complete mesocolic excision (CME)^20,29–31^, improves significantly the oncological outcomes, with 5-year cancer-related survival to 89% and a local recurrence rate less than 4%, after R0 resections^20,29,30^.

In our study we investigated oncological outcomes in 3 groups of patients, all treated for splenic flexure cancer between January 2006 and May 2016, comparing segmental resection versus extended right or left hemicolectomy, to search for the best oncological surgical approach for splenic flexure cancer.

At our knowledge, this is the first study that compare three different surgical approaches for splenic flexure cancer with a so large cohort of patients. Despite this is a monocentric study, the statistic analysis including 103 patients appears adequate, confirming the oncological feasibility of the segmental resection compared with extended resections, with similar oncologic quality of resection and postoperative outcomes.

In the searched literature, almost all studies about splenic flexure cancer surgery compare the right extended colon resection versus the left extended colon resection, with poor data concerning the long-term oncological outcomes^25,32,33^.

These studies suggest that the extent of the surgical procedure does not influence the quality of resection or the postoperative outcomes and report that 77% of patients with SFC were treated with left colectomy^32^.

Our results showed no significant differences in the oncological outcomes between the three groups. Stage of disease according to AJCC/UICC TNM were similar. In terms of surgical quality surrogates, the number of harvested lymphnodes and R0 rate were similar in the 3 groups. The proportion of patients with more than 12 harvested lymphnodes was also not significantly different.

The higher number of harvested lymphnodes in the ERH group, was probably associated to larger extension of resected colon with least 3 colonic vascular pedicles, as also reported by other authors^32^.

According to Perrakis *et al*., the rationale for extended resection associated to splenectomy and/or distal pancreasectomy, is the potential metastatic lymphnodes along with the right gastroepiploic arcade at the greater curvature of the stomach, over the pancreatic head and along the inferior aspect of the pancreas^34^. We believe that the motivation for extensive resections appears to failin front of comparable R0 margin rate and oncologic outcomes, and that a resection extended to near organs is mandatory only in real case of tumor infiltration. In our series, a multiorgan resection was performed in 11 patients, but in only 4,8% of patients we found a T4 disease.

As far as complication rates, no technique seems safer than others. No significant differences in complications according to severity, reoperation rate, hospital stay, 30-day mortality, were found among three groups.

Looking at the long-term survival outcomes, the type of procedure was not a significant predictor, with no significant differences among the three groups.

Our results suggest that a segmental splenic flexure resection is oncologically adequate for splenic flexure carcinoma. As in all colon cancer surgery a correct CME procedure, including a sharp dissection along embryological planes and achieving a specimen with intact mesocolic fasciae which envelope the lymphatic drainage of the tumor is mandatory. Furthermore, the resection for splenic flexure carcinoma includes foremost the left colic and secondly the left branch of the middle colic lymphoadenectomy, guaranteeing the removal of the mostly involved lymphatic drainage of a splenic flexure cancer.

## Conclusion

Despite the limitations of a retrospective study, our results provide valuable support for the oncological adequacy of a segmental resection of splenic flexure cancer. The R0 margin and a lymphoadenectomy with at least 12 harvested lymphnodes together with the surgical specimen, are the foundation of a correct surgical procedure, independently from the extension of the resection. Complete mesocolic excision is the way to achieve an optimal lymphnode yield. Hence, the surgical strategy in terms of extension of colonic resection seems not to have an influence on the final stage classification and the survival.

## References

[1] Steffen C, Bokey EL, Chapuis PH (1987). Carcinoma of the splenic flexure. Dis Colon Rectum..

[2] Levien DH, Gibbons S, Begos D, Byrne DW (1991). Survival after resection of carcinoma of the splenic flexure. Dis Colon Rectum..

[3] Shaikh IA (2012). Does the outcome of colonic flexure cancers differ from the other colonic sites?. Int J Colorectal Dis..

[4] Nakagoe T (2000). Carcinoma of the splenic flexure: multivariate analysis of predictive factors for clinicopathological characteristics and outcome after surgery. J Gastroenterol..

[5] Bourgouin S (2012). Three-dimensional determination of variability in colon anatomy: applications for numerical modeling of the intestine. J Surg Res..

[6] Odermatt M (2014). Short- and long-term outcomes for patients with splenic flexure tumours treated by left versus extended right colectomy are comparable: a retrospective analysis. Surg Today..

[7] Milone, M. *et al*. *Surg Endosc*. **17**, 3467–3473 (2018).

[8] Pisani Ceretti A. (2015). Laparoscopic colonic resection for splenic flexure cancer: our experience. BMC Gastroenterol..

[9] Chong CS (2016). Operative Method for Transverse Colon Carcinoma: Transverse Colectomy Versus Extended Colectomy. Dis Colon Rectum..

[10] Kim CW, Shin US, Yu CS, Kim JC (2010). Clinicopathologic characteristics, surgical treatment and outcomes for splenic flexure colon cancer. Cancer Res Treat..

[11] van Rongen I, Damhuis RA, van der Hoeven JA, Plaisier PW (2013). Comparison of extended hemicolectomy versus transverse colectomy in patients with cancer of the transverse colon. Acta Chir Belg..

[12] Rega D (2016). Carcinoma of the splenic flexure: What surgical treatment. EJSO..

[13] Clavien PA (2009). The Clavien-Dindo classification of surgical complications: five-year experience. Ann Surg..

[14] Dindo D, Demartines N, Clavien PA (2004). Classification of surgical complications: a new proposal with evaluation in a cohort of 6336 patients and results of a survey. Ann Surg..

[15] UICC TNM classification of malignant tumors, 7^th^ edn. John Wiley & Sons, New York (2009).

[16] https://www.aiom.it/linee-guida/linee-guida-aiom-2018-tumori-del-colon/ (2018)

[17] Feig, B. W. & Ching, C. D. The MD Anderson Surgical Oncology Handbook, Department of Surgical Oncology. (eds University of Texas MD Anderson Cancer Center) (Philadelphia, P. A.: Lippicott Williams & Wilkins 2011).

[18] Kim JW (2016). Short- and long-term outcomes of laparoscopic surgery vs open surgery for transverse colon cancer: a retrospective multicenter study. Onco Targets Ther..

[19] Sakorafas GH, Zouros E, Peros G (2006). Applied vascular anatomy of the colon and rectum: clinical implications for the surgical oncologist. Surg Oncol..

[20] West NP (2012). Understanding optimal colonic cancer surgery: comparison of Japanese D3 resection and European complete mesocolic excision with central vascular ligation. J Clin Oncol..

[21] Otchy D (2004). Standards Practice Task Force; American Society of Colon and Rectal Surgeons. Practice parameters for colon cancer. Dis Colon Rectum..

[22] Griffiths JD (1956). Surgical anatomy of the blood supply of the distal colon. Ann R Coll Surg Engl..

[23] Nakagoe T (2001). Surgical treatment and subsequent outcome of patients with carcinoma of the splenic flexure. Surg Today.

[24] Vasey CE, Rajaratnam S, O'Grady G, Hulme-Moir M (2018). Lymphatic Drainage of the Splenic Flexure Defined by Intraoperative Scintigraphic Mapping. Dis Colon Rectum..

[25] de'Angelis N (2016). Laparoscopic extended right colectomy versus laparoscopic left colectomy for carcinoma of the splenic flexure: a matched case-control study. Int J Colorectal Dis..

[26] Shen SS (2009). Number of lymph nodes examined and associated clinicopathologic factors in colorectal carcinoma. Arch Pathol Lab Med..

[27] Dotan E, Cohen SJ (2011). Challenges in the management of stage II colon cancer. Semin Oncol..

[28] Wolpin BM, Mayer RJ (2008). Systemic treatment of colorectal cancer. Gastroenterology..

[29] Weber K, Merkel S, Perrakis A, Hohenberger W (2013). Is there a disadvantage to radical lymph node dissection in colon cancer?. Int J Colorectal Dis..

[30] West NP (2010). Complete mesocolic excision with central vascular ligation produces an oncologically superior specimen compared with standard surgery for carcinoma of the colon. J Clin Oncol..

[31] Chang GJ, Rodriguez-Bigas MA, Skibber JM, Moyer VA (2007). Lymph node evaluation and survival after curative resection of colon cancer: systematic review. J Natl Cancer Inst..

[32] Martínez-Pérez A (2017). Surgical Treatment of Colon Cancer of the Splenic Flexure: A Systematic Review and Meta-analysis. Surg Laparosc Endosc Percutan Tech..

[33] Gravante G (2016). Extended right hemicolectomy and left hemicolectomy for colorectal cancers between the distal transverse and proximal descending colon. Ann R Coll Surg Engl..

[34] Perrakis A (2014). Lymph node metastasis of carcinomas of transverse colon including flexures. Consideration of the extramesocolic lymph node stations. Int J Colorectal Dis..

